# Passive smoking exposure and incidence and disease outcomes of inflammatory bowel disease: a systematic review and meta-analysis

**DOI:** 10.3389/fpubh.2025.1670320

**Published:** 2025-10-29

**Authors:** Akanksha Mahajan, Bhawna Gupta, Adam Peterson, Guru Iyngkaran, Zina Valaydon

**Affiliations:** ^1^Monash Health, Melbourne, VIC, Australia; ^2^Department of Public Health, Torrens University, Melbourne, VIC, Australia; ^3^Gastroenterology Department, Monash Health, Melbourne, VIC, Australia; ^4^School of Clinical Sciences at Monash Health, Monash University, Melbourne, VIC, Australia; ^5^Department of Gastroenterology, Royal Melbourne Hospital, Parkville, VIC, Australia; ^6^Department of Gastroenterology, Western Health, Footscray, VIC, Australia

**Keywords:** inflammatory bowel disease, ulcerative colitis, passive smoking, smoking, tobacco, pregnancy, Crohn’s disease, second hand smoke

## Abstract

**Introduction:**

Inflammatory bowel disease includes a range of chronic gastrointestinal disorders, most commonly Crohn’s Disease (CD) and ulcerative colitis (UC). This systematic review and meta-analysis aims to evaluate the effect of passive smoking on incidence and disease outcomes of CD and UC.

**Methods:**

This review was conducted according to the Preferred Reporting Items for Systematic Review and Meta-Analysis (PRISMA) guidelines. Meta-analysis was performed according to the Meta-analysis of Observational Studies in Epidemiology (MOOSE) guidelines. The literature was systematically searched from inception until May 2025 to identify relevant studies from a range of databases including MEDLINE, CINAHL, Embase, Scopus and Cochrane Library.

**Results:**

The initial search yielded 151 articles, with 32 studies deemed relevant for inclusion. Significant associations with passive smoking exposure were seen in 8 out of 20 studies for increased risk of CD and 3 out of 17 studies for UC. Meta-analysis found that passive smoking during childhood (OR 1.19, 95% CI 1.05–1.35) and exposure to smoking during pregnancy (OR 1.27, 95% CI 1.03–1.55) was associated with increase in odds of CD; however neither exposure was associated with an increased odds of UC. Associations with CD were also not confirmed in sensitivity analysis of higher-quality studies. Passive smoking was associated with disease complications including pouch-itis and backwash-ileitis in UC; while exposure to smoking during pregnancy was associated with hospitalisation and colorectal neoplasia in CD. There is inconclusive evidence surrounding the effects of passive smoking on need for medications and surgery.

**Discussion:**

Findings of this review highlight the importance of educating on harms of passive smoking.

**Systematic review registration:**

https://www.crd.york.ac.uk/PROSPERO/view/CRD420251035510.

## Introduction

Inflammatory bowel disease (IBD) is comprised of a group of chronic relapsing–remitting inflammatory disorders of the gastrointestinal tract. IBD is broadly classified into Crohn’s disease (CD) and ulcerative colitis (UC) which differ in location, histology and clinical phenotype (1). IBD presents a growing global burden of disease, with increasing prevalence of cases, deaths and DALYs (disability adjusted life years). A total of 147 out of 204 countries or territories studied in the Global Burden of Disease database demonstrated an increase in the age-standardised prevalence rate of IBD from 1990 to 2019 (2). While IBD has conventionally been considered a disease of Western nations; over the past three decades, there has been a rising incidence in newly industrialised countries in Africa, Asia, and South America while prevalence remains high in Europe, North America and Oceania (3). Moreover, there is compounding prevalence of IBD in Western countries, because even though the incidence is stabilising, new diagnoses of IBD continue to increase the prevalent population of IBD patients (4). Due to natural population growth over time, there is forecasted to be doubling of prevalence from 0.5 to 1.0% from 2008 to 2030 in some regions of North America and Europe (5). This change in disease epidemiology in line with the westernisation of countries with historically low rates of IBD supports that environmental factors are involved in disease pathogenesis (6). This environmental influence is further supported by a Canadian study by Benchimol et al. (7) which found that offspring of immigrants from low-incidence regions have a similar risk of acquiring IBD as individuals in Western populations.

The pathogenesis of IBD is multifactorial with an interplay between genetic susceptibility and environmental factors which drives dysregulation in immune response, intestinal epithelial barrier dysfunction and an altered gut microbiome leading to an abnormal immune response to intestinal flora (6, 8–10). The interaction between multiple pathogenic factors in the environment, genome, microbiome and immunological factors leads to a network effect which triggers disease. This is referred to as the ‘IBD interactome’ (10). Although family history is the greatest risk factor for developing IBD, genetic susceptibility does not explain the variance in disease incidence (11, 12). Modifiable risk factors which have been implicated in the pathogenesis of IBD include smoking, poor sanitation, air and water pollution, diet, food additives and antibiotic use (13). There is growing evidence to suggest that the dynamic balance between gut flora, and host defensive responses in intestinal mucosal epithelium has a pivotal role in the initiation of IBD (6).

This intestinal inflammation is associated with heterogenous clinical presentations with abdominal pain, diarrhoea, and bloody stools; as well as extra intestinal manifestations and systemic symptoms such as weight loss and fatigue (14). IBD is associated with significant morbidity, with associated hospitalisations and surgeries leading to substantial healthcare costs and burden on healthcare systems (15–17). There are also extensive indirect costs through loss of productivity, with a Swedish cost-effectiveness modelling study finding that these accounted for around 50% of disease-associated costs (4).

Although active smoking is an established risk factor for CD (18), there remains controversy surrounding its influence on the development and course of UC. Studies have shown that smoking can downregulate apical tight junction protein genes, leading to increased epithelial paracellular permeability which triggers cellular apoptosis, mucosal erosion and ulcers that can contribute to intestinal epithelium damage (19, 20). Moreover, IBD has been associated with an imbalance in the composition of host-associated microbiota and a shift towards potentially pathogenic microorganisms; known as dysbiosis (21). Cigarette smoking can cause a shift in microbiome composition and activity in the colon; as evidenced in a Korean population-based study which found altered faecal microbiota composition in current smokers compared to those who had never smoked (20, 22). Similarly, a study on patients undergoing endoscopy for upper GI symptoms, iron deficiency or Crohn’s disease found that there was reduced bacterial diversity in the upper small intestinal mucosa of current smokers as compared to never smokers (23). Moreover, chronic cigarette smoking is associated with impaired gas exchange and consequently systemic and colonic tissue ischemia which drives intestinal epithelial barrier dysfunction (24).

The role of passive smoking in both CD and UC is underexplored. During cigarette combustion, accumulated chemicals are distributed among mainstream smoke that is inhaled into the lungs during the puffs, side stream smoke that is released by the burning tip of the cigarette, secondhand smoke that is a mixture of sidestream and exhaled mainstream smoke, and thirdhand smoke which is the toxic tobacco residue found on surfaces exposed to smoke, as well as the ashes and cigarette butts (25). Hence, both smokers and non-smokers in the presence of smokers are exposed to known toxic compounds in cigarette smoke, including nicotine, heavy metals, nitrosamines, phenyls and insecticides (25). Approximately 37% of the population globally is exposed to the smoke emitted from the burning end of tobacco products or exhaled from smokers; known as passive smoking; with higher rates of exposure among women and children compared to men (26, 27). In 2021, 2,709 million people were exposed to passive smoking, and the five countries with the largest populations exposed were China, India, Indonesia, Pakistan and United States (28). Children are particularly vulnerable to the harmful effects of passive smoking, as they are involuntarily exposed to this for years, if living with family members who smoke at home. The Global Burden of Disease Study 2019 estimates that 50,000 deaths and 4,500,000 disability-adjusted life-years among children under 14 years of age were attributable to passive smoking (29).

This systematic review aims to evaluate the effect of passive smoking in early life (prenatal and during childhood, i.e. <18 years of age) on incidence and disease outcomes of CD and UC. This is the first review to investigate the effect of passive smoking on disease outcomes in IBD.

## Methods

This study is registered at the International Register of Prospective Systematic Reviews (PROSPERO) under CRD420251035510. This review was conducted according to the Preferred Reporting Items for Systematic Review and Meta-Analysis (PRISMA) guidelines.

### Eligibility criteria

All observational studies that evaluated effect of passive smoking exposure during gestation or childhood (age <18 years) on incidence of CD or UC were included. Passive smoking exposure during gestation refers to maternal active or passive smoking during pregnancy. Patients diagnosed with CD or UC at any age, and in any healthcare setting were considered. Patients diagnosed with Inflammatory Bowel Disease Unclassified or microscopic colitis were excluded from this review.

### Search strategy

The literature was systematically searched from inception until May 2025 to identify relevant studies from a range of databases including MEDLINE, CINAHL, Embase, Scopus and Cochrane Library. An English translation was obtained for any articles not published in English. The following key words were used: ‘Passive smok*’, ‘second hand smok*’, ‘inflammatory bowel disease’, ‘Crohn disease’ and ‘ulcerative colitis’; combined with Boolean operators. This search was supplemented with a manual search of the reference lists of relevant articles as well as grey literature such as conference abstracts and posters.

### Data extraction

Search data were uploaded to the Endnote software, and duplicates were removed. Two reviewers (AM and BG) independently screened the title and abstract of the identified citations. Full texts were subsequently independently screened by two reviewers (AM and BG) for eligibility. Disagreements were resolved by discussion among the authors with a consensus decision being reached. Data was extracted from eligible studies including year of publication, study design, sample size, definition of exposure and outcome, inclusion and exclusion criteria, adjusted covariates, measures of association including both unadjusted and adjusted odds ratios. Outcome data related to cases of CD, cases of UC, presence of dose response relationship, risk of complicated B2 (stricturing) or B3 (penetrating) disease as per the Montreal Classification of IBD (30), need for biologics, hospitalisation risk, number of cases requiring immunosuppression, number requiring steroid therapy, number requiring surgery; and incidence of colorectal neoplasia were collected. If separate measures of association were reported with regards to sources of passive smoking exposure (e.g., in the home, from mother, father, other etc.), then the most inclusive measure of association was used. Passive smoking exposure during childhood was defined as exposure to smoking within the household or elsewhere from any persons, below the age of 18 years. Passive smoking exposure during pregnancy was defined as maternal smoking during any stage of pregnancy.

### Quality assessment

The quality of studies was assessed by two authors (AM and BG), with any disagreements resolved by consensus. Quality assessment was completed using the Newcastle-Ottawa Scale, which contains eight items, divided into three categories: selection, comparability, and depending on the study type, outcome (cohort studies) or exposure (case–control studies). The scoring ranges from one to nine stars, as a maximum of one star can be assigned per item with the exception of the item related to comparability which allows for two stars (31). Scoring is as follows:

- Good quality = 3–4 stars in selection AND 1–2 stars in comparability AND 2–3 stars in outcome.
- Fair quality = 2 stars in selection AND 1–2 stars in comparability AND 2–3 stars in outcome.
- Poor quality = 0–1 star in selection OR 0 star in comparability OR 0–1 star in outcome (31).

### Statistical analysis

The meta-analysis was performed according to the Meta-analysis of Observational Studies in Epidemiology (MOOSE) guidelines. Review Manager (RevMan) version 7.2.0 was used to conduct all statistical analyses. Forest plots were generated to demonstrate the effect of each study and the pooled effect size for studies with the same outcome. A probability value of *p* < 0.05 was considered statistically significant. A random effects model using the DerSimonian and Laird method was applied for meta-analysis as it incorporates heterogeneity of treatment effects between studies into the analysis (32). Unadjusted odds ratio (OR) along with their corresponding 95% CI were used as the effects of measure for dichotomous outcomes related to incidence and/or complications associated with inflammatory bowel disease. The authors of this review manually calculated the unadjusted OR when it was not included in a publication, but adequate data was provided for calculation.

For each overall effect size, heterogeneity was examined using Cochran’s Q statistic (measure of weighted square deviations), with *N* − 1 degrees of freedom (where *N* is the number of studies), between studies variance (T2), and ratio of the true heterogeneity to total observed variation (*I*^2^). Potential causes for heterogeneity were evaluated through sensitivity analysis. Publication bias was assessed through visual inspection of funnel plots.

## Results

### Search results and study characteristics

**Figure 1:**
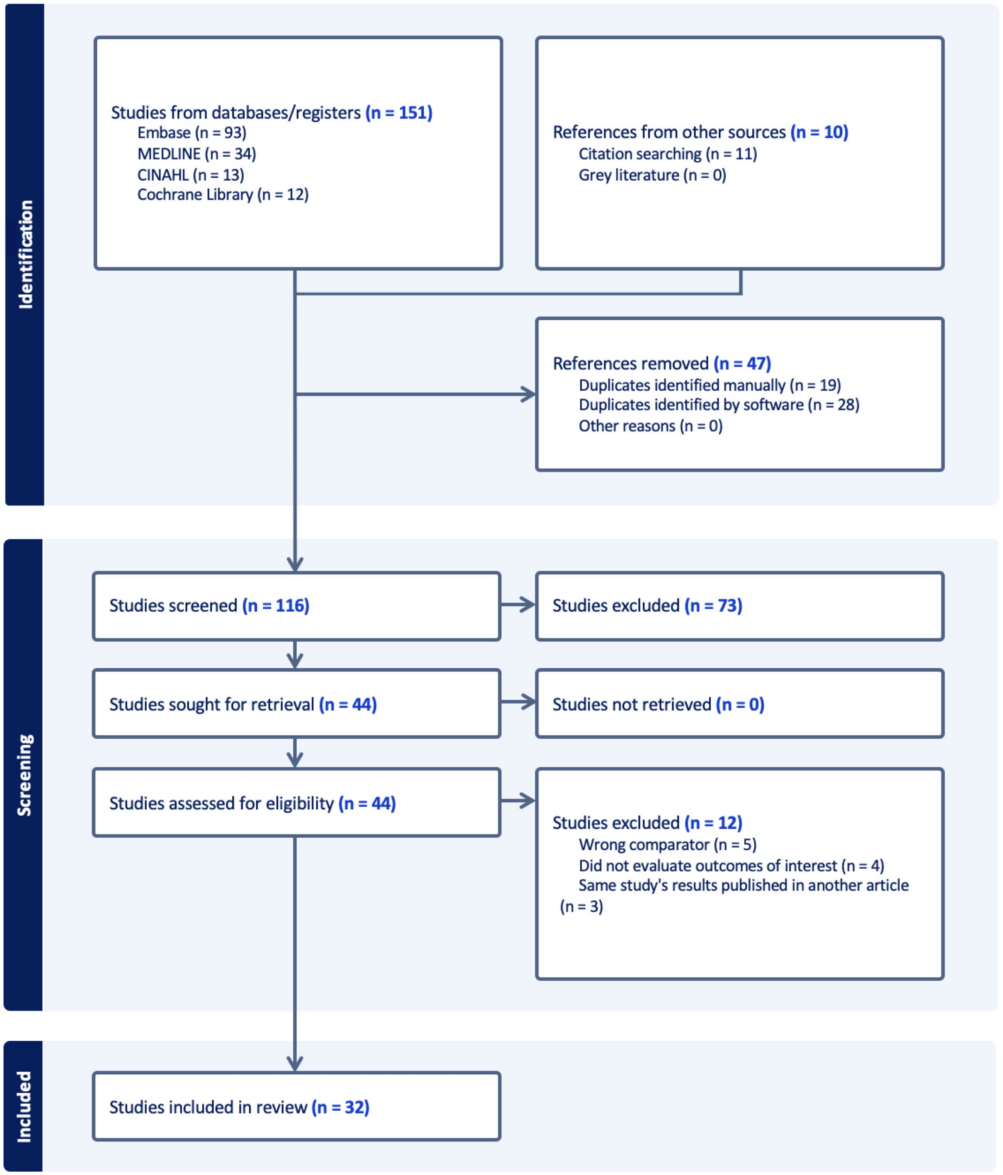
PRISMA flowchart.

The PRISMA flow diagram in Figure 1 shows that the initial search yielded 151 articles. Following the removal of duplicates, the title and abstracts of 114 articles, and subsequently full texts of 42 articles, were reviewed by 2 authors (AM and BG), with 32 studies deemed relevant for inclusion. This review includes 25 studies which evaluated the effect of passive smoking on incidence of CD and UC, with 21 case control studies, 3 prospective cohort studies, and one Mendelian randomisation study. It includes 6 cohort studies which assessed the effect of passive smoking on complications of CD (33–38), and 4 studies on complications of UC, of which there were 3 cohort studies (36–38) and 1 cross sectional study (39). Included studies were of varying quality; with 11 high quality studies (33, 35, 38, 40–47), 5 of fair quality (48–52), and 16 poor quality studies (34, 36, 37, 39, 53–63); as detailed in Supplementary Table 1. Quality of the study by Yu et al. (64) was not evaluated using the Newcastle Ottawa Scale as this scale is not applicable to Mendelian randomisation studies.

This systematic review includes studies across a range of geographic locations, with 11 studies from Europe (5 from United Kingdom, 2 from Sweden, 1 from Sweden and Norway, and 1 each from France, Finland and the Netherlands), 5 studies from Asia (3 from Japan, 1 each from Qatar and Israel), and 7 from North America (2 from Canada and 5 from the United States of America). However, there was only one study conducted in Africa and South America respectively, and none conducted in Oceania that were eligible for inclusion in this review. The studies included were all conducted in industrialised countries, except for 1 study in Brazil which is an emerging region. Hence, the review may not be representative of the entire global population of IBD patients. Although this may be partially attributable to the low incidence of IBD in South America and Africa, incidence of IBD in these regions is rising alongside increasing industrialisation, and there is a poor understanding of this evolving epidemiology which should be addressed with future research (18, 65). There is also a need for further research on this subject in Oceania, which has longstanding high incidence and prevalence rates of IBD (66).

### Effects of passive smoking on incidence of IBD

A total of 25 studies, with 81,257 patients with CD and 5,349 patients with UC were included in the section of the review on incidence of IBD. Significant associations with passive smoking exposure were seen in 8 out of 20 studies for increased risk of CD and 3 out of 17 studies for UC. Interestingly, a Mendelian randomisation study by Yu et al. found a significant association between workplace exposure to passive smoking and incidence of UC, but not CD (64). Meanwhile, a case control study by Eliakim et al. (53) found no association between passive smoking at work and either CD or UC. A dose response relationship was reported in 2 studies for passive smoking exposure during childhood on incidence of CD (50, 63) and 1 for UC (51). Similarly, another study found that greater number of years of living with a smoker was associated with slightly increased odds of developing CD (44). Studies also reported that higher number of cigarettes smoked during pregnancy (52) or in the household during childhood (56) increased risk of IBD; although associations with CD and UC were not reported separately.

**Figure 2:**
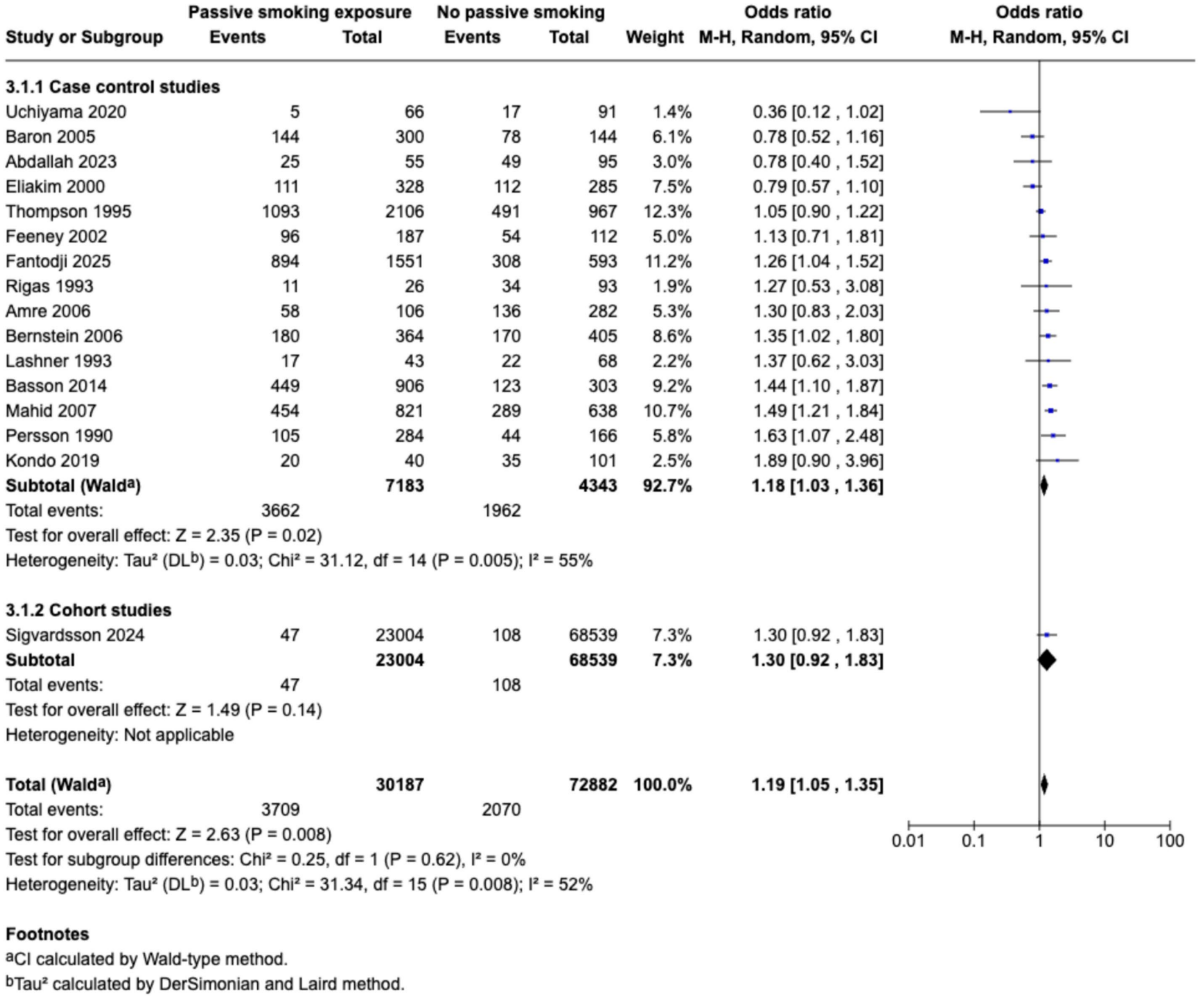
Passive smoking exposure and risk of CD.

**Figure 3:**
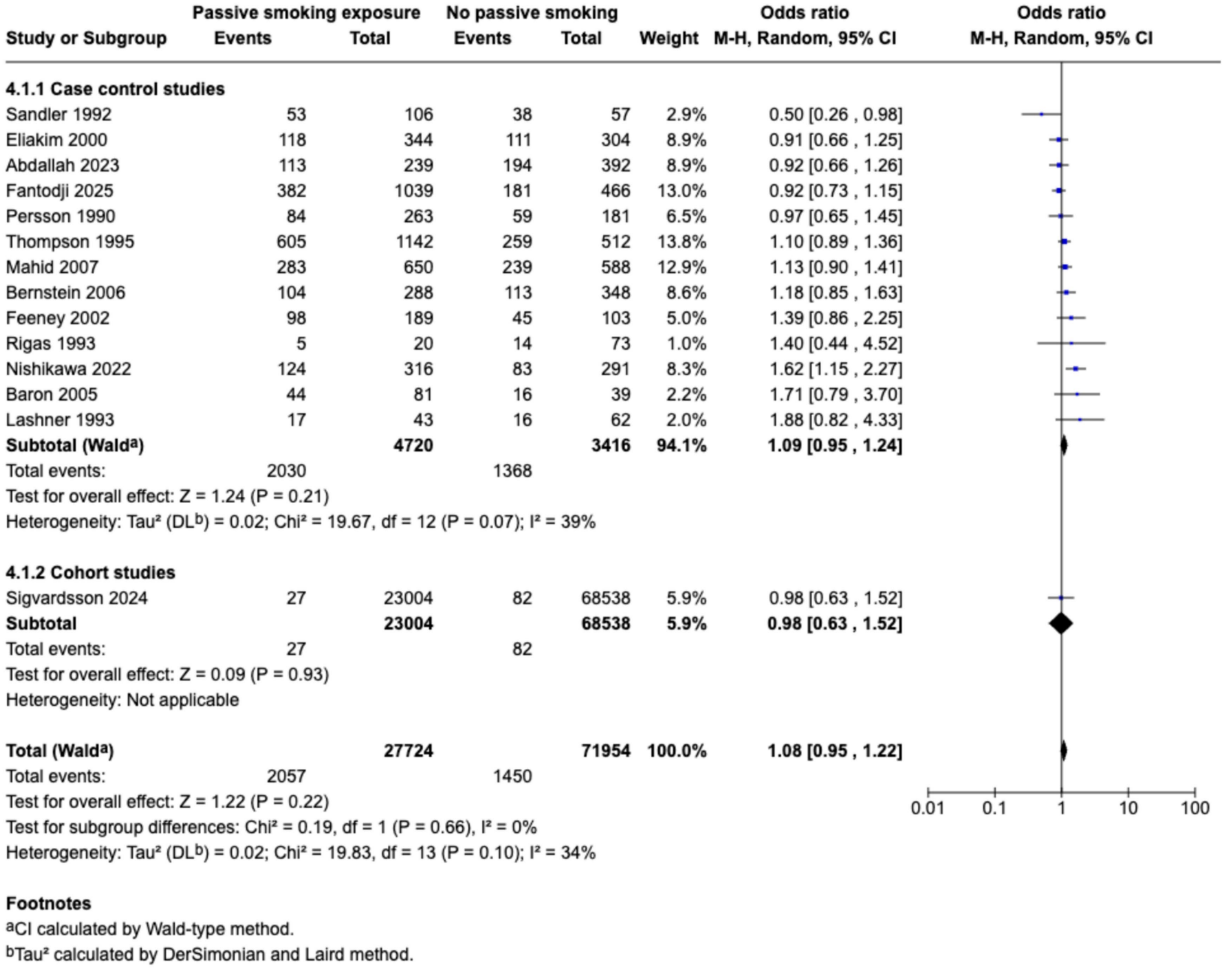
Passive smoking exposure and risk of UC.

**Figure 4:**
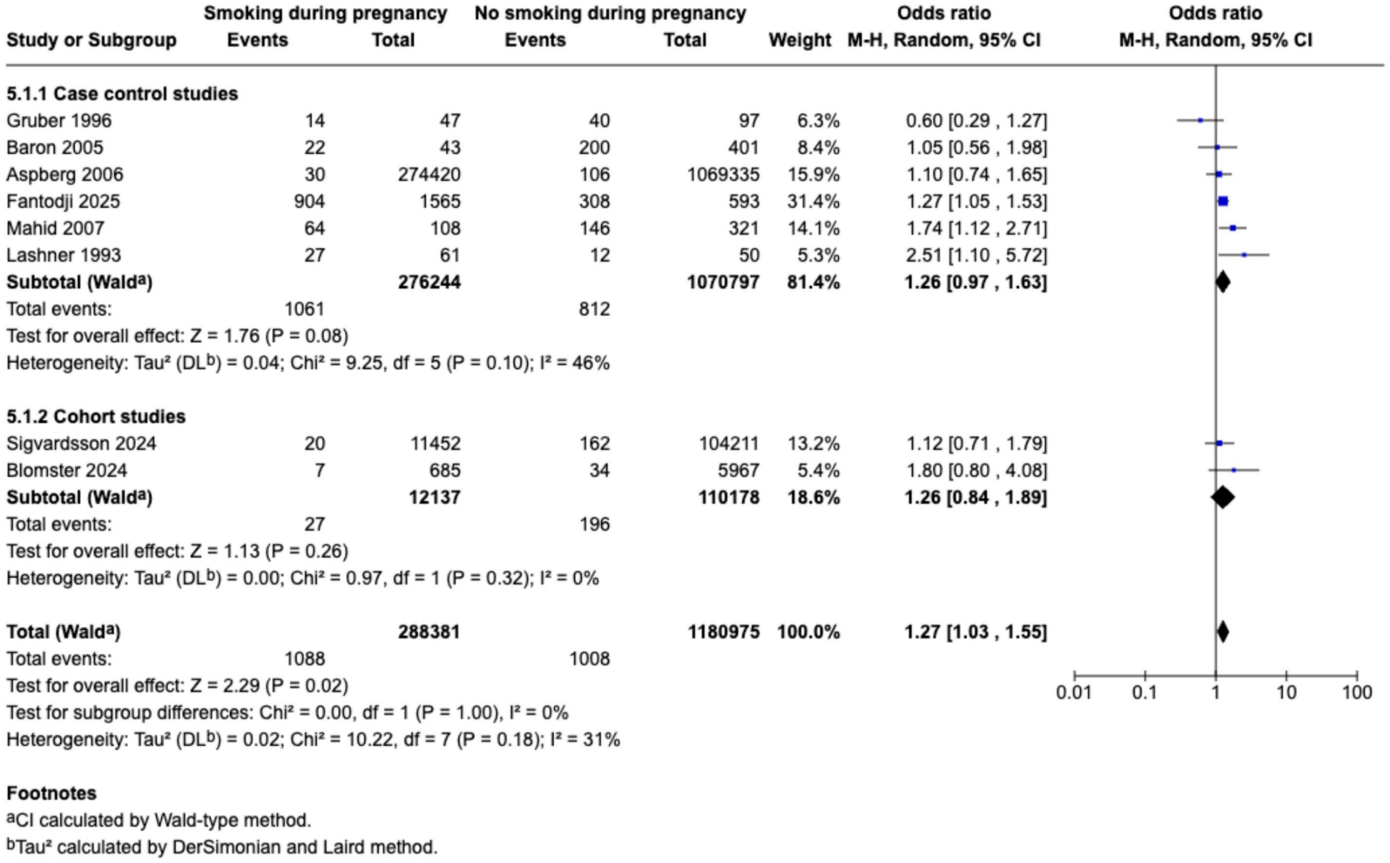
Maternal smoking during pregnancy and odds of CD in offspring.

**Figure 5:**
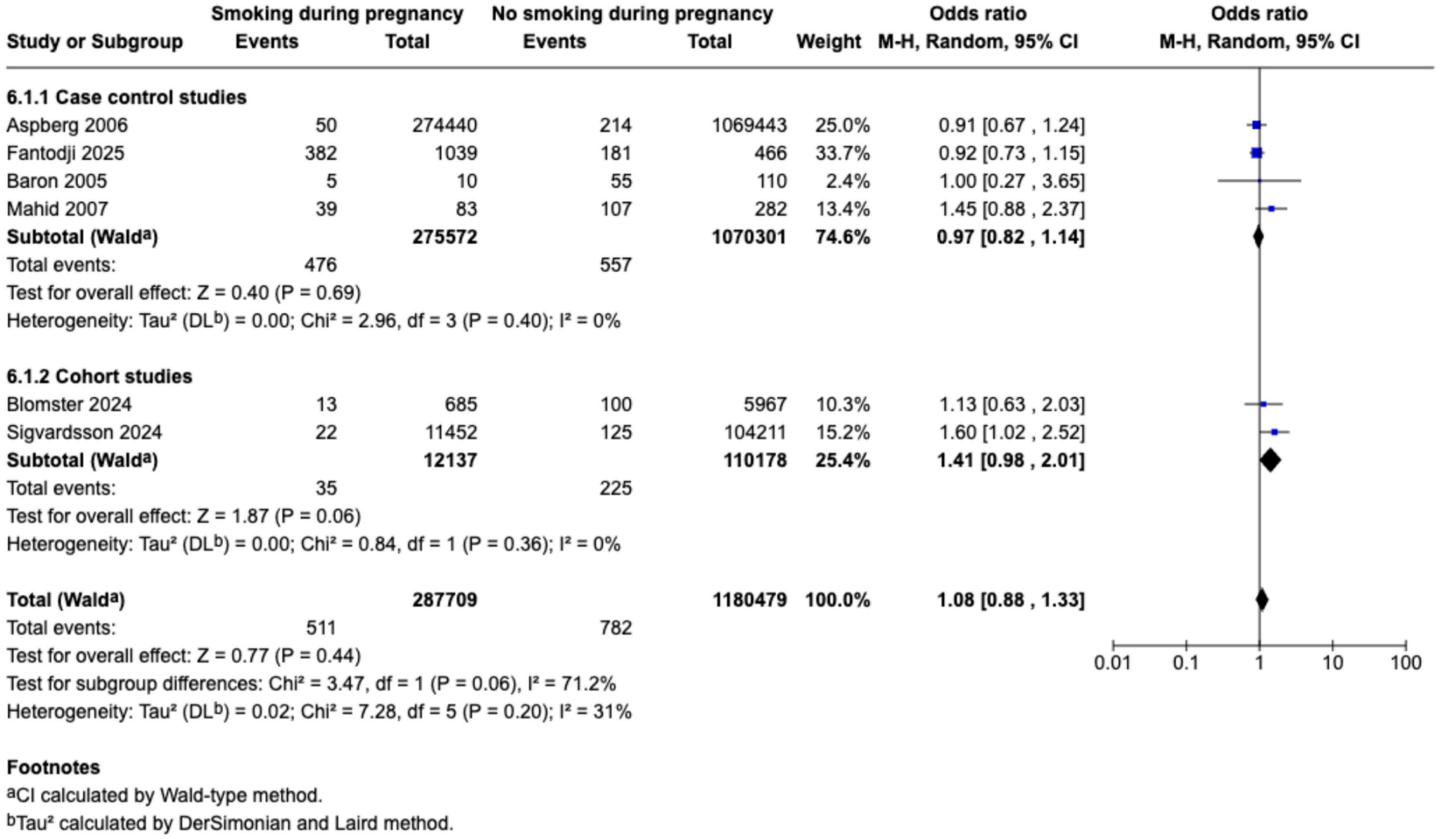
Maternal smoking during pregnancy and odds of UC in offspring.

Meta-analysis found that passive smoking during childhood (OR 1.19, 95% CI 1.05–1.35) and exposure to passive smoking in utero via maternal active or passive smoking, (OR 1.27, 95% CI 1.03–1.55) was associated with increase in odds of CD; however, neither exposure was associated with incidence of UC; as demonstrated in the forest plots in Figures 2–5. Sensitivity analysis which only included studies of high quality, found that there was no significant association between passive smoking during pregnancy or life and incidence of either CD or UC.

Funnel plots for studies evaluating association between childhood passive smoking exposure as well as during pregnancy for both CD and UC had a symmetrical distribution, suggesting that publication bias was not present, as shown in Supplementary Figures 1–4.

### Effects of passive smoking on complications of IBD

A total of 7 studies, with 2,962 patients with CD and 1,238 patients with UC were included in the review of effect of passive smoking on disease outcomes of IBD. Meta-analysis was not performed due to a limited number of studies reporting on each outcome of interest, as well as heterogeneity across the included studies. Two studies found that passive smoking exposure was not significantly associated with disease outcomes of CD (33, 34), but passive smokers with UC were significantly more likely to develop pouchitis (inflammation of the surgically created ileal pouch) after undergoing ileoanal pouch anastomoses surgery, and backwash ileitis (36). Four studies evaluated risk of hospitalisations associated with passive smoking in patients with CD (33, 34, 36, 37), while 3 studies evaluated this for UC (36, 37, 39). Although one study found that exposure to maternal smoking during pregnancy was significantly associated with hospitalisation in CD (33), no significant association was found between passive smoking during life and hospitalisation for CD or UC in any of the included studies.

However, there is inconclusive evidence surrounding the effects of passive smoking on need for medications and surgery. While one study found a positive association between requirement for immunosuppression with anti-tumour necrosis factor-α monoclonal antibody infliximab and passive smoking exposure for CD (36); others did not find any significant association for immunosuppression (34) or steroid use (34, 36) in CD or UC (36). Contrasting outcomes were also observed with respect to risk of surgery. One study found it was associated with increased likelihood of intestinal surgery in CD (35), whilst another found no association with either CD and UC (36). Interestingly, passive smoke exposure was associated with increased risk of colorectal neoplasia in CD but not UC (38).

## Discussion

This systematic review and meta-analysis found that exposure to passive smoking during pregnancy and childhood is associated with an increased incidence of CD but not UC. This observation is supported by a positive dose response relationship between increasing number of cigarettes smoked and risk of IBD. However, the association was not identified following a sensitivity analysis for only high quality studies, and reflects the conclusion of Jones et al. who reported no association between childhood passive smoke exposure and CD or UC (67).

**Figure 6:**
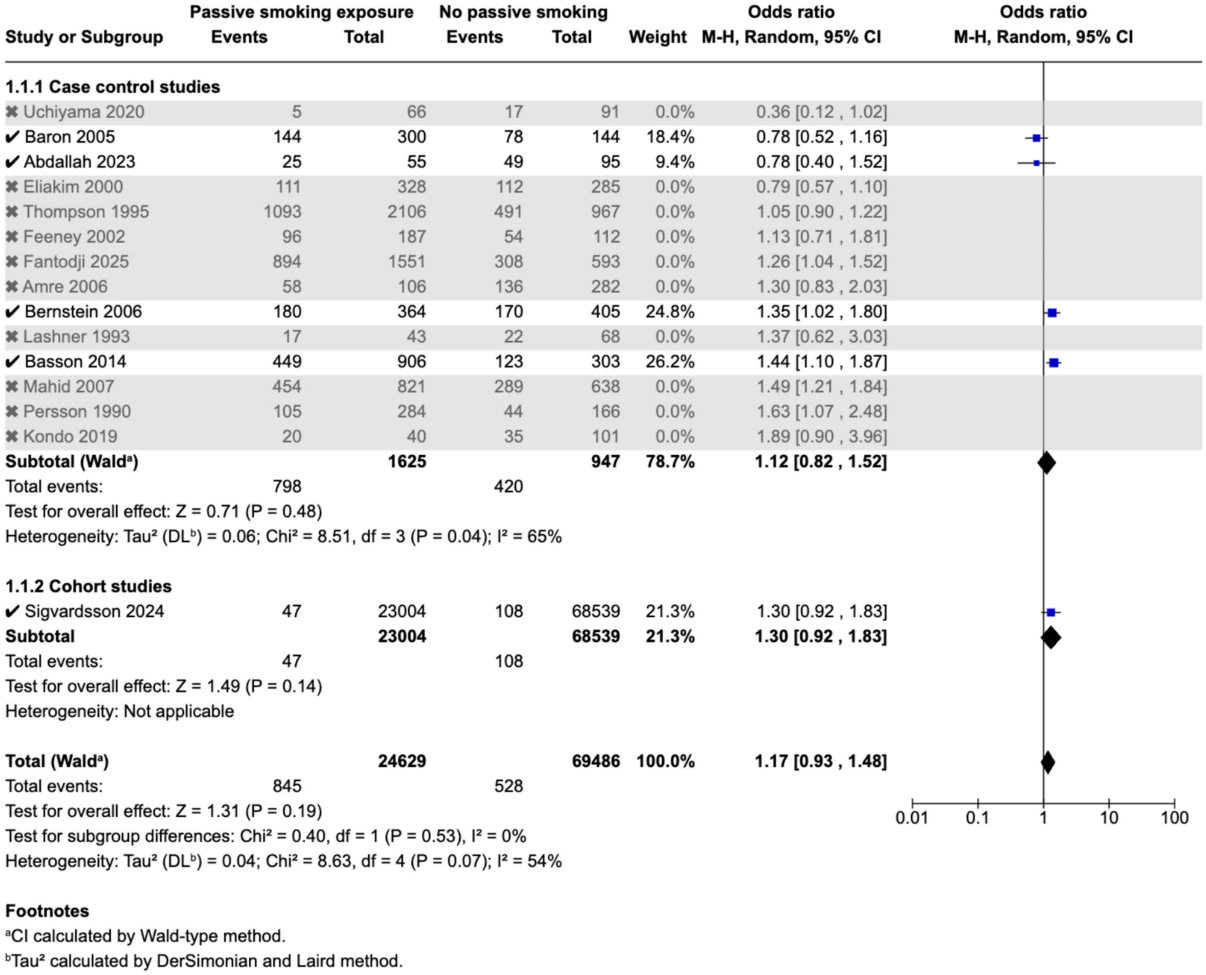
Sensitivity analysis including only high quality studies for passive smoking during childhood and CD.

**Figure 7:**
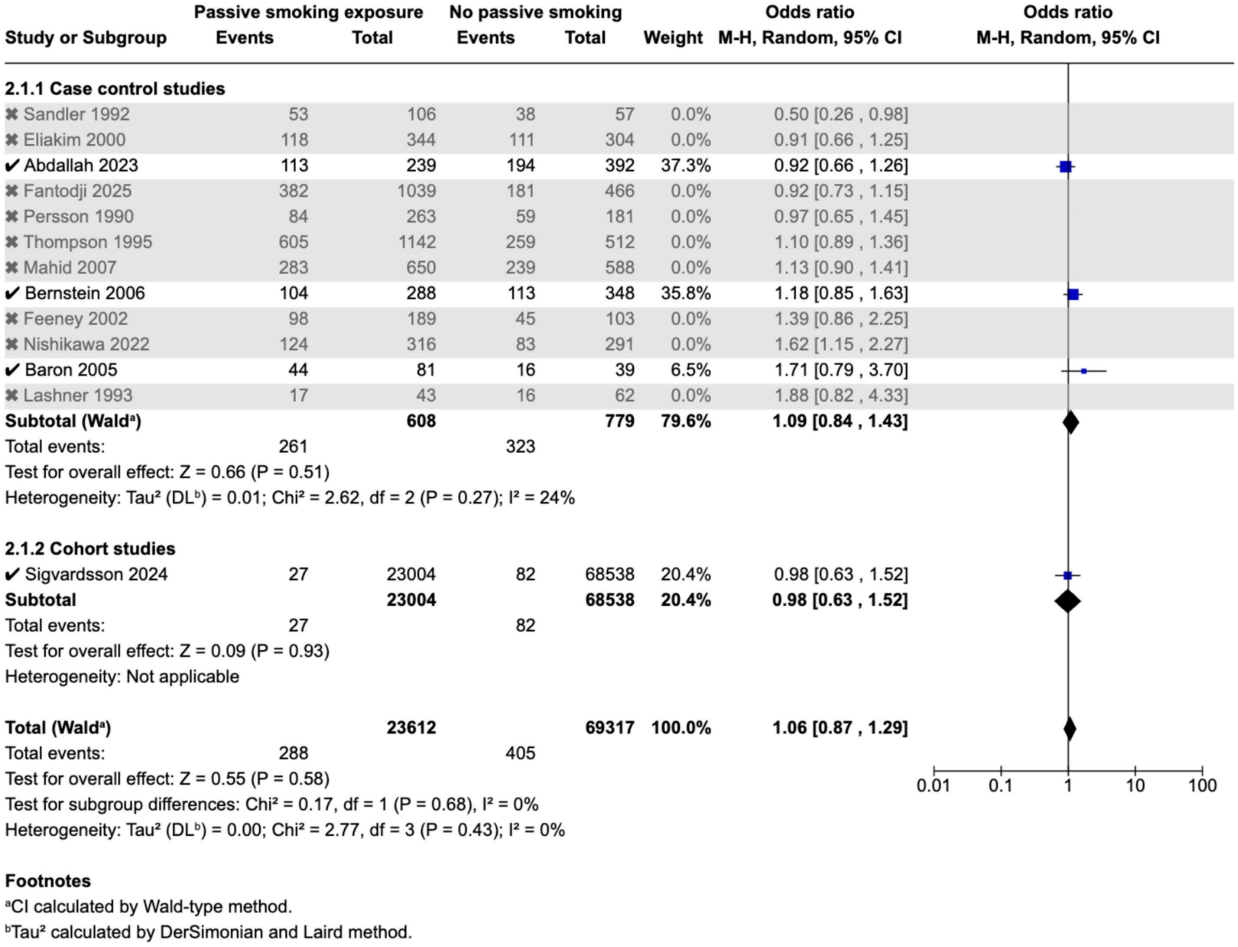
Sensitivity analysis including only high quality studies for passive smoking during childhood and UC.

**Figure 8:**
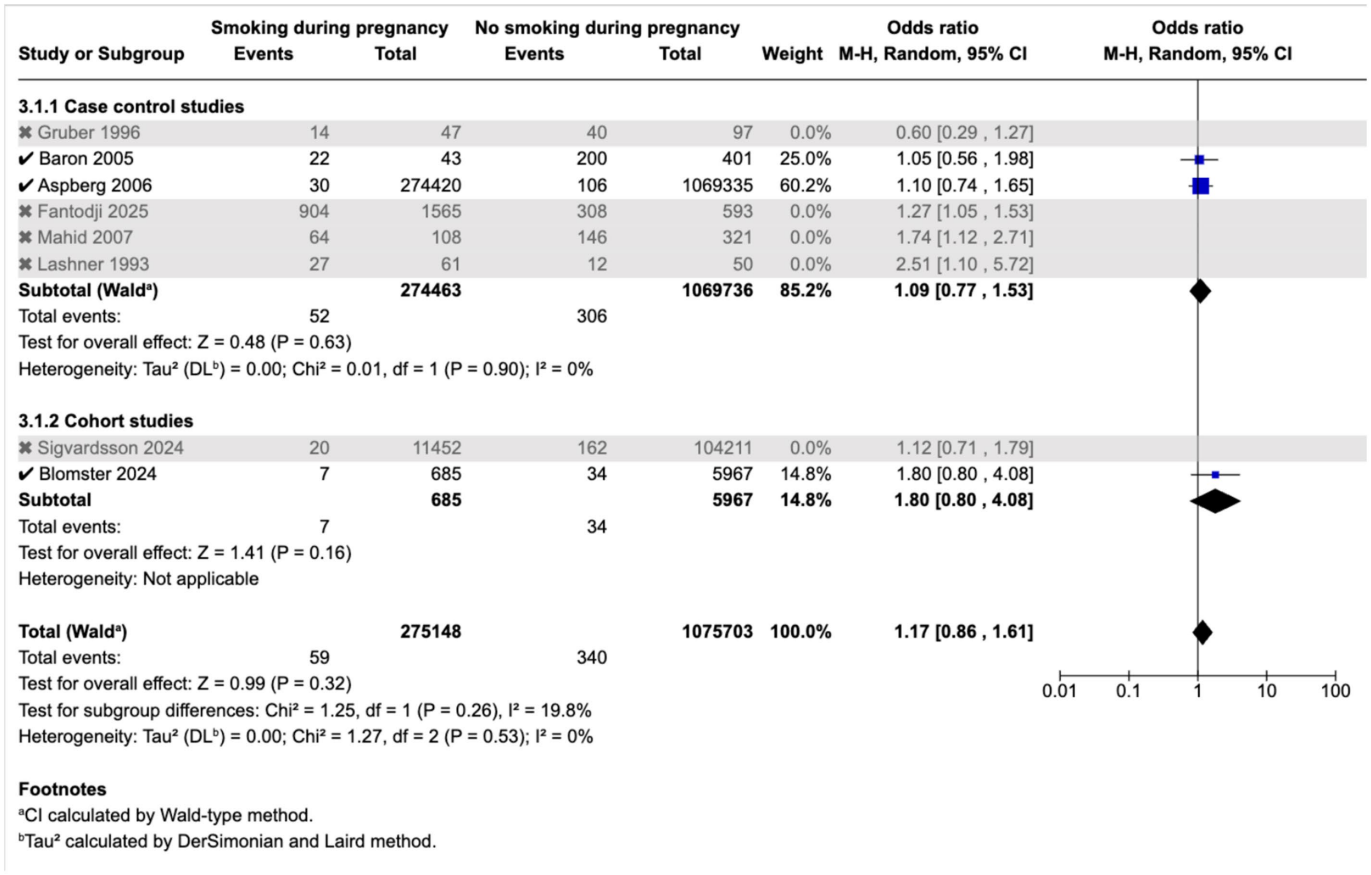
Sensitivity analysis including only high quality studies for maternal smoking during pregnancy and CD.

**Figure 9:**
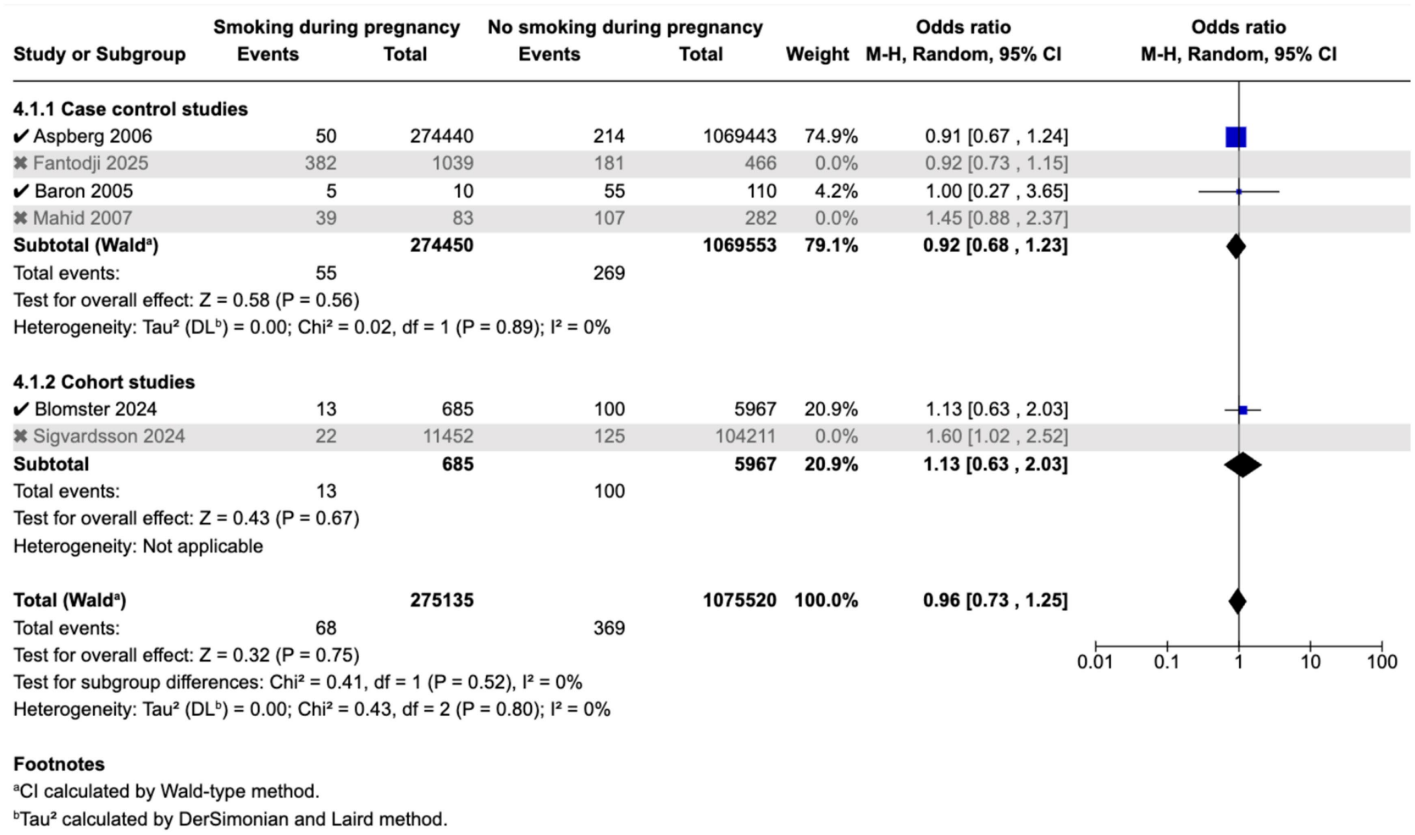
Sensitivity analysis including only high quality studies for maternal smoking during pregnancy and UC.

It is possible that the association was not seen in the sensitivity analysis due to reduced statistical power from a smaller sample size. Importantly, an increased risk of IBD was observed in persons exposed to passive smoking, in the study by Basson et al. (43), which was the largest of the high quality studies (Figures 6–9).

Mechanistic animal studies support the biological plausibility of the impact of passive smoke exposure on risk of IBD. Tobacco smoke has been shown to alter mucous composition in murine models (68), alter the human microbiota (69), and impair intestinal epithelial barrier function in murine small bowel (70). Although interestingly, the colon was not affected. Similarly, smoking increases inflammatory cell recruitment and pro-inflammatory cytokine release (71). However, tolerogenic effects have been observed in UC as compared to CD and may explain differences in epidemiological outcomes for these patients. Passive smoke exposure has been specifically examined in a rat model of trinitrobenzene sulfonic acid-induced experimental colitis, where exposure to 2% or 4% cigarette smoke-filled air resulted in increased colonic oedema, mucosal lesion area, and histopathological score. Mucosal myeloperoxidase activity and tumour necrosis factor α level as markers of colitis activity were also elevated (72). Passive smoking exposure has also been shown to significantly increase the expression of intestinal α7 nicotinic acetylcholine receptors (α7nAChRs) which play a key role in the cholinergic anti-inflammatory pathway (72). α7nAChR is upregulated in UC and CD intestinal tissue (73), however some preclinical studies have shown that it may have a protective role and its significance remains unclear (74–76).

The effect of smoking and indeed passive smoke exposure on UC is less clear than in CD, and our review found no significant association with incidence of UC. There was no association between passive smoking and UC complications except for pouchitis and backwash- ileitis, which are inherently disorders of the small bowel rather than colon. Although this is the first review to study the effects of passive smoking on IBD complications; our findings are corroborated by previous meta-analyses which found that active smoking was not associated with surgery or flare of disease activity in UC (77, 78). One previous review has explored the effects on incidence of IBD (67), however this paper includes a much larger number of studies and consequently greater sample population, leading to increased reliability and representativeness of conclusions.

There are a number of reasons why passive smoking may more likely contribute to development of CD but not UC. Cigarette smoke has been associated with increased apoptosis in follicle-associated epithelium overlying Peyer’s patches in mouse models as well as an upregulation in mRNA expression of CCL9 and CCL20, two important chemokines in CD pathogenesis (79). This epithelial barrier damage may lead to exposure of lumen antigens, which can be recognized by immune cells whose recruitment is increased, inducing the development of CD (80).

Moreover, a murine study demonstrated that cigarette smoke exposure damages the ileal mucosal barrier, generating greater permeability to bacteria—this modification to gut microbiota is likely to more significantly influence development of CD whereas development of UC has greater association with changes in humoral immunity (80). Additionally, the immunomodulatory and anti-inflammatory effects of nicotine and carbon monoxide may predominate in UC but not in ileal CD, due to the different expression of receptors and other molecules by immune cells residing in the different parts of the intestine that are affected in CD or UC (80). The immunomodulatory effect of nicotine is mediated by the activation of α7-nAChR in immune cells which, although having controversial and multifactorial mechanisms of actions, has been found to decrease production of pro-inflammatory cytokines, and contribute to immunosuppressive function of CD4+ CD25+ regulatory T cells, reducing NF-κB activation and IL-2 production (81). Carbon monoxide, the main component of the gas phase of cigarette smoke, has also been associated with reduced production of pro-inflammatory cytokines.

Smoking is an independent risk factor for colorectal neoplasia (CRN) (82), however only a single study by Van der Sloot et al. (38) assessed this risk in IBD. Whilst there was an association with previous smoking and CRN, there was no significant association found between passive smoking and CRN in patients with UC (38). This finding may be attributable to recall bias given that this was a retrospective cohort study or may suggest a dose response whereby passive smoking has not provided sufficient exposure to the toxins and carcinogens present in cigarettes as compared to active smoking. Further studies are required to corroborate the results of this single study.

Our review found some evidence to support a dose response relationship between passive smoking exposure during childhood and pregnancy and incidence of UC and CD (44, 45, 50–52, 56, 63). However, given the small sample size and uneven distribution across different degrees of smoking exposure during childhood/pregnancy in the existing literature, there is a need for further research to more rigorously examine this.

This review highlights the importance of health policies and public health interventions targeted at cessation of smoking, particularly at home. Several interventions have been implemented in recent years to address this. There is evidence to support that smoke-free regulations such as the smoke-free outdoor regulation across the WHO European Region are effective in reducing second hand smoking exposure when extensive or complete smoking bans were implemented (83, 84). However, partial bans are less effective in reducing second hand smoke exposure (84). Hence, there is a role for stricter legislation regarding smoking in public places. Follow-up data from monitoring and evaluation of the efficacy of and compliance to these interventions for second hand smoking reduction is essential to guide future laws and policy. For instance, the Tobacco Control Scale (85) has been used to research the effectiveness of tobacco control policy in the European Union annually. However, these programs to reduce smoking in public places will not address exposure in the home and further education may be required in order to address the association between passive smoking during pregnancy and childhood and incidence of IBD identified in this review. Smoking is also more prevalent in lower socioeconomic groups which already have higher morbidity from chronic disease; hence interventions targeting smoking cessation and education regarding the risks of second hand smoke for these vulnerable populations may be effective in preventing the exacerbation of existing health inequities (86, 87).

Early smoking cessation education and intervention for patients and more importantly families, to address risk factors such as passive smoking is crucial to reduce economic strain of IBD management. For instance, a study conducted in Australia on resource use for management of IBD found that inpatient IBD management and treating active disease was associated with significantly higher costs compared to management of outpatients and disease in remission, respectively (15, 17). Proactive care may help prevent disease from reaching a severity where reactive and resource-intensive management is required such as hospital admission and treatment with biologics (15, 16).

## Limitations

All of the included studies measured passive smoking exposure through self-reported data from IBD patients, except for the cohort study by Nowak et al. in which presence and concentration of cotinine in urine was used an objective marker of smoking exposure. Thus, the data is subjected to reporting bias as well as recall bias; especially given that most included studies were retrospective. There is a particular risk for social desirability bias and consequent falsely low exposure reporting given the social unacceptability and stigma surrounding smoking during pregnancy and around children, which may have led to parents not disclosing this exposure to IBD patients, or hesitation in accurate reporting by patients themselves for fear of judgement. Moreover, many of the included case–control studies did not specify how CD or UC was diagnosed in cases, or measures to determine that controls definitely did not have IBD (i.e., symptom free or previous colonoscopy); which further reduces reliability of findings. Disease complications and their definitions were also not standardised across included studies, and this did not allow for meta-analyses to be performed for these outcomes.

Additionally, only one of the included studies reported duration of maternal smoking during pregnancy or specific number of years of passive smoking exposure during childhood; which limits the ability to draw conclusions regarding potentially varying impacts of passive smoking exposure at different points in gestation or evaluation of a dose response relationship with respect to duration of exposure.

## Conclusion

This review suggests a possible link between childhood passive smoking and Crohn’s disease, but this was not confirmed in sensitivity analysis of higher-quality studies. No consistent associations were found for ulcerative colitis or disease outcomes. These results should be interpreted cautiously, and further high-quality prospective studies examining the effect of passive smoking on disease outcomes are required for both CD and UC.

## Data Availability

The original contributions presented in the study are included in the article/Supplementary material, further inquiries can be directed to the corresponding authors.
