# Correction: Passive smoking exposure and incidence and disease outcomes of inflammatory bowel disease: a systematic review and meta-analysis

**DOI:** 10.3389/fpubh.2026.1815067

**Published:** 2026-03-06

**Authors:** Akanksha Mahajan, Bhawna Gupta, Adam Peterson, Guru Iyngkaran, Zina Valaydon

**Affiliations:** 1 Monash Health, Melbourne, VIC, Australia; 2 Department of Public Health, Torrens University, Melbourne, VIC, Australia; 3 Gastroenterology Department, Monash Health, Melbourne, VIC, Australia; 4 School of Clinical Sciences at Monash Health, Monash University, Melbourne, VIC, Australia; 5 Department of Gastroenterology, Royal Melbourne Hospital, Parkville, VIC, Australia; 6 Department of Gastroenterology, Western Health, Footscray, VIC, Australia

**Keywords:** inflammatory bowel disease, ulcerative colitis, passive smoking, smoking, tobacco, pregnancy, Crohn's disease, second hand smoke

Author Bhawna Gupta was erroneously not marked as an additional corresponding author.

The original version of this article has been updated.

