# Supplementary material for: Passive smoking exposure and incidence and disease outcomes of inflammatory bowel disease: a systematic review and meta-analysis
Source: Front Public Health. 2025 Oct 29;13:1670320. doi: 10.3389/fpubh.2025.1670320 (PMC12605268; doi:10.3389/fpubh.2025.1670320)

Supplementary figure 1: Funnel plot for passive smoking exposure in childhood and incidence of Crohn’s disease


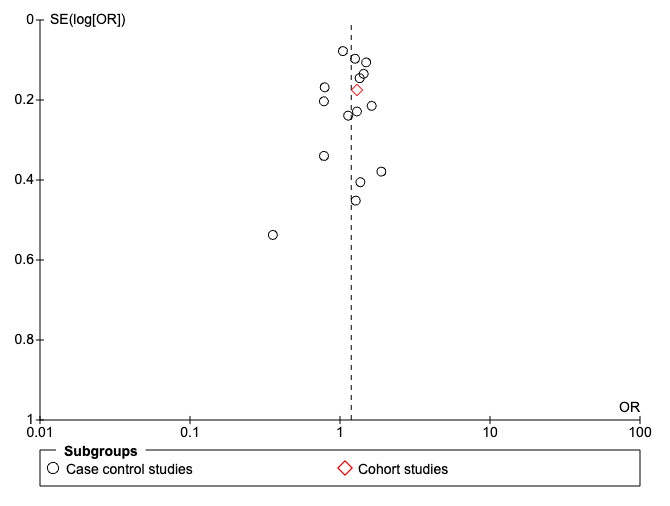


Supplementary figure 2: Funnel plot for passive smoking exposure in childhood and incidence of UC


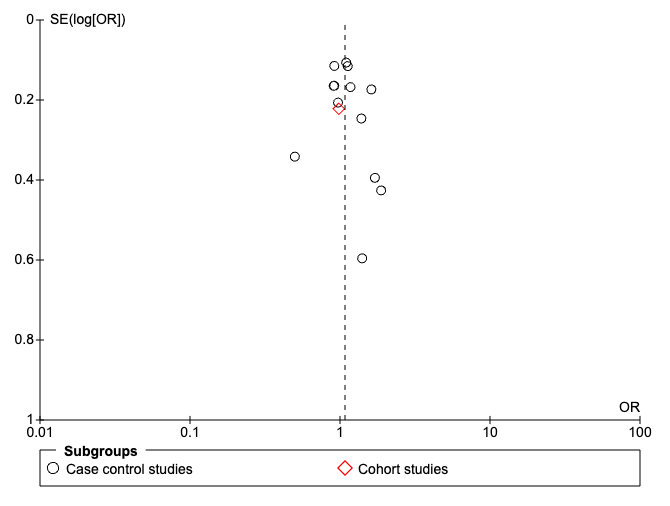


Supplementary figure 3: Funnel plot for passive smoking exposure during pregnancy and incidence of Crohn’s disease


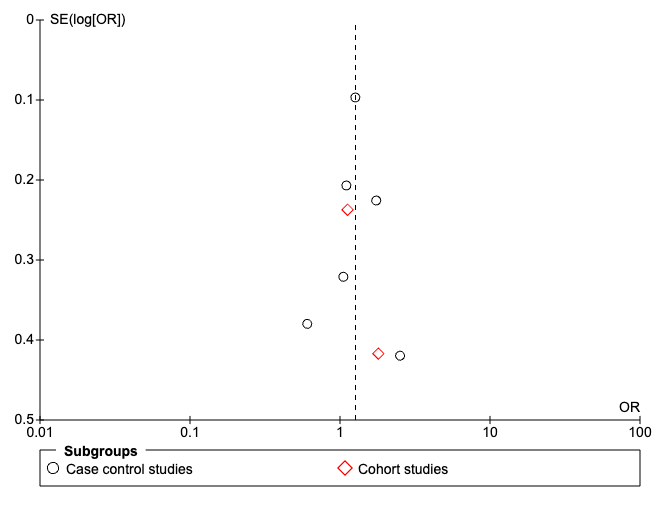


Supplementary figure 4: Funnel plot for passive smoking exposure during pregnancy and incidence of UC


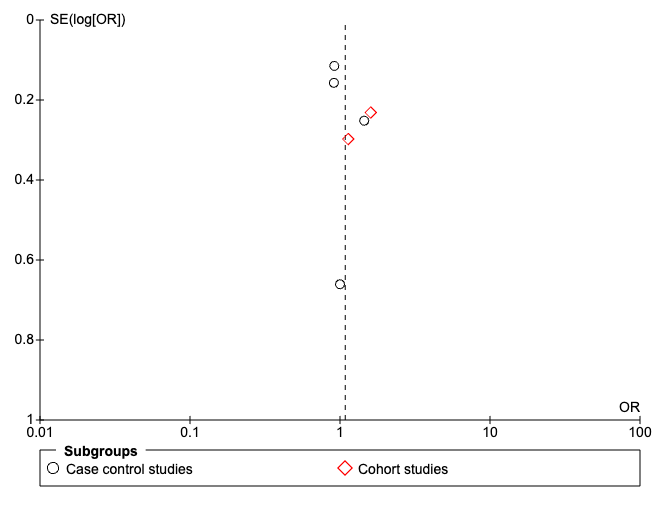

Supplement: Supplementary file 1 [file Data_Sheet_1.docx]
