# Supplementary material for: Passive smoking exposure and incidence and disease outcomes of inflammatory bowel disease: a systematic review and meta-analysis
Source: Front Public Health. 2025 Oct 29;13:1670320. doi: 10.3389/fpubh.2025.1670320 (PMC12605268; doi:10.3389/fpubh.2025.1670320)
Supplement: Supplementary file 2 [file Table_1.docx]

Supplementary Table 1: Quality assessment of included studies based on the Newcastle Ottawa Scale

| **Reference** | **Overall** | **Selection** | | | | **Comparability** | **Outcome/Exposure** | | |
| --- | --- | --- | --- | --- | --- | --- | --- | --- | --- |
|  | | **Accurate case definition (case control)/demonstration that outcome not present at start of study (cohort)** | **Representativeness of cases (case control)/exposed (cohort)** | **Selection of controls (case control)/non-exposed (cohort)** | **Definition of controls (case control)/demonstration that outcome of interest was not present at start of study (cohort)** | **Comparability of groups** | **Ascertainment of exposure (case control)/assessment of outcome (cohort)** | **Non response rate (case control)/adequacy of follow up (cohort)** | **Same ascertainment method (case control)/ duration of follow up (cohort)** |
| Abdallah, 2023 | good, 8 | 1 | 1 | 1 | 1 | 2 | 0 | 1 | 1 |
| Amre, 2006 | fair, 7 | 1 | 1 | 0 | 0 | 2 | 1 | 1 | 1 |
| Aspberg, 2006 | good, 8 | 1 | 1 | 1 | 1 | 2 | 0 | 1 | 1 |
| Baron, 2005 | good, 8 | 1 | 1 | 1 | 1 | 2 | 0 | 1 | 1 |
| Basson, 2014 | good, 8 | 1 | 1 | 0 | 1 | 2 | 1 | 1 | 1 |
| Bernstein, 2006 | good, 8 | 1 | 1 | 1 | 1 | 2 | 0 | 1 | 1 |
| Blomster, 2024 | good, 8 | 1 | 1 | 1 | 1 | 2 | 0 | 1 | 1 |
| Eliakim, 2000 | poor, 5 | 0 | 1 | 1 | 0 | 0 | 1 | 1 | 1 |
| Fantodji, 2025 | fair, 7 | 1 | 1 | 1 | 1 | 2 | 0 | 0 | 1 |
| Feeney, 2002 | poor, 4 | 1 | 0 | 0 | 1 | 0 | 1 | 0 | 1 |
| Gruber, 1996 | poor, 5 | 0 | 0 | 1 | 1 | 2 | 0 | 0 | 1 |
| Hu, 2024 | good, 8 | 1 | 1 | 1 | 1 | 2 | 0 | 1 | 1 |
| Kondo, 2019 | fair, 7 | 1 | 1 | 0 | 1 | 2 | 0 | 1 | 1 |
| Lashner, 1993 | poor, 6 | 1 | 1 | 1 | 0 | 2 | 0 | 0 | 1 |
| Lindoso, 2018 | good, 8 | 1 | 1 | 1 | 1 | 2 | 0 | 1 | 1 |
| Mahendraratnam, 2009 | poor, 3 | 1 | 0 | 1 | 0 | 0 | 0 | 0 | 1 |
| Mahid, 2007 | poor, 5 | 1 | 0 | 0 | 1 | 2 | 0 | 0 | 1 |
| Martins, 1996 | poor, 4 | 0 | 0 | 0 | 1 | 2 | 0 | 0 | 1 |
| Nishikawa, 2022 | fair, 6 | 1 | 0 | 0 | 1 | 2 | 0 | 1 | 1 |
| Nowak 2008 | poor, 3 | 1 | 0 | 0 | 0 | 0 | 1 | 0 | 1 |
| Persson 1990 | poor, 5 | 0 | 1 | 0 | 1 | 2 | 0 | 0 | 1 |
| Russel, 2005 | poor, 2 | 0 | 0 | 0 | 0 | 2 | 0 | 0 | 0 |
| Sandler, 1992 | poor, 4 | 0 | 0 | 1 | 0 | 2 | 0 | 0 | 1 |
| Scharrer, 2021 | good, 8 | 1 | 1 | 1 | 1 | 2 | 0 | 1 | 1 |
| Sigvardsson, 2024 | fair, 7 | 1 | 1 | 1 | 1 | 2 | 0 | 0 | 1 |
| Thompson, 1995 | poor, 2 | 0 | 0 | 1 | 0 | 0 | 0 | 0 | 1 |
| Uchiyama, 2020 | poor. 6 | 1 | 0 | 1 | 1 | 1 | 0 | 1 | 1 |
| van der Heide, 2009 | poor, 6 | 1 | 1 | 1 | 1 | 0 | 0 | 1 | 1 |
| van der Heide, 2011 | poor, 6 | 1 | 1 | 1 | 1 | 0 | 0 | 1 | 1 |
| van der Sloot, 2020 | good, 8 | 1 | 1 | 1 | 1 | 2 | 0 | 1 | 1 |
| van der Sloot, 2022 | good, 8 | 1 | 1 | 1 | 1 | 2 | 0 | 1 | 1 |
| Yu, 2025 | n/a | n/a | n/a | n/a | n/a | n/a | n/a | n/a | n/a |
